# *SCN8A* mutations in Chinese patients with early onset epileptic encephalopathy and benign infantile seizures

**DOI:** 10.1186/s12881-017-0460-1

**Published:** 2017-09-18

**Authors:** Jiaping Wang, Hua Gao, Xinhua Bao, Qingping Zhang, Jiarui Li, Liping Wei, Xiru Wu, Yan Chen, Shujie Yu

**Affiliations:** 1 Department of Pediatrics, Peking University First Hospital, Beijing, 100034 China; 2 Center for Bioinformatics, State Key Laboratory of Protein and Plant Gene Research, School of Life Sciences, Peking University, Beijing, China; 3 Department of Neurology, Harbin Children’s Hospital, Harbin, Heilongjiang Province 150010 China

**Keywords:** *SCN8A*, Epileptic encephalopathy, Family cases

## Abstract

**Background:**

*SCN8A* mutations have recently been associated with epilepsy and neurodevelopmental disorders. This study aimed to broaden the phenotypic-spectrum of disease related with *SCN8A* mutations.

**Methods:**

To identify the pathogenic gene of a Chinese family, in which six members suffered from epilepsy, whole-exome sequencing was performed. In addition, target next-generation sequencing (NGS) was performed on 178 sporadic patients, who had epilepsy of unknown etiology within 6 months after birth. A detailed clinical history was obtained.

**Results:**

A heterozygous missense mutation of *SCN8A* was identified in the Chinese family. Six de novo mutations of *SCN8A* were detected in 6 sporadic patients with epilepsy. In the family, six members developed seizures within a few years after birth. Five of them had milder clinical performance, that they had normal cognition and developmental milestones, and seizure-free was achieved by mono-therapy. The other one affected member presented with refractory epilepsy and developmental regression. She died from sudden unexpected death in epilepsy (SUDEP) at 17-year-old. Clinical features of six sporadic patients with *SCN8A* mutations were diverse, ranging from severe epileptic encephalopathy to benign epilepsy with normal cognition. Seizures started at the mean age of 3.9 months (from 2 months to 6 months). Seizure-free was achieved in four of them by mono- or multi-antiepileptic drugs. Five of them demonstrated mild or severe psychomotor retardation, whereas the other one was normal in development and intelligence.

**Conclusions:**

Our findings extend the spectrum of *SCN8A* mutations and the clinical features of patients with *SCN8A* mutations. The majority of *SCN8A* mutations were de novo, inherited mutations from the heterozygous parents can also occur. The phenotypic spectrum of *SCN8A* mutation varied largely. Most affected patients manifested as refractory epilepsy and severe intellectual disability, only a small number of patients presented with milder clinical patterns. Additionally, our study confirmed that the same mutation can lead to different phenotypes.

**Electronic supplementary material:**

The online version of this article (10.1186/s12881-017-0460-1) contains supplementary material, which is available to authorized users.

## Background

Voltage-gated sodium channels (VGSCs) play a critical role in controlling neuronal excitability. VGSCs contain one pore-forming α-subunit and one or two β subunits. The α-subunit is made up of four repeated homologous domains (I ~ IV), each domain containing a motif of six transmembrane segments (S1 ~ S6) and an additional pore loop located between the S5 and S6 segments [1]. So far, 9 VGSCs α-subunits (Na_v_1.1 ~ Na_v_1.9) have been identified. Among these 9 α-subunits, Na_v_1.1, 1.2, 1.3 and 1.6, which are encoded by *SCN1A*, *SCN2A*, *SCN3A* and *SCN8A* respectively, are primarily expressed in the central nervous system. Mutations in these genes can result in epilepsy [2–5].

The Na_v_1.6 α-subunit is encoded by *SCN8A*, which is highly expressed along the axonal initial segment (AIS) and the nodes of Ranvier of myelinated axons [6, 7]. Na_v_1.6 is directly involved in action potential generation and conduction. Mutations of *SCN8A* are associated with type 13 of early infantile epileptic encephalopathy (EIEE 13) (OMIM #614558) [8, 9]. The phenotypic spectrum of diseases caused by *SCN8A* mutations varied largely. Most patients had intractable epilepsy beginning at the first year of life, accompanied by severe developmental delay and intellectual disability. *SCN8A* mutations can also lead to milder phenotype, such as benign infantile seizures (BFIS)/infantile convulsion and paroxysmal choreoathetosis (ICCA) [10]. Part of patients with *SCN8A* mutations presented only mental retardation without epilepsy [11]. In this study, seven *SCN8A* mutations were identified in a Chinese family and six sporadic patients with unknown etiological epilepsy, including four previously described mutations and three *novel* mutations.

## Methods

### Patients and samples

**Fig. 1:**
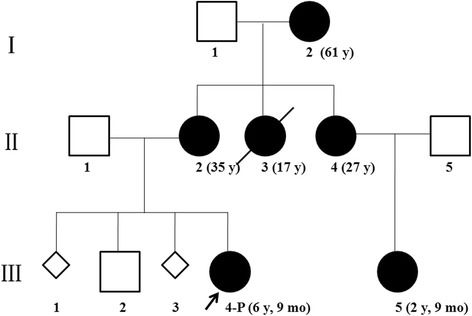
The pedigree of family case with *SCN8A* mutation.I-2, II-2, II-4, III-2, III-4, were analyzed by whole-exome sequencing. III-5 was tested by Sanger sequencing

A Chinese family was recruited in this study, in which six members spanning 3 generations were affected with epilepsy (Fig. 1). All of them were female, and aged from 2 to 61 years old. *PCDH19* gene mutation was ruled out in this family. The proband had focal seizures at the age of 6 months. Seizure frequency ranged from one time per month to 6 times per day. She was treated with valproic acid (VPA) at 8 months of age. Seizure-free was achieved at 1 year old. III-5 had similar clinical course as the proband. Seizures attacked at 6-month-old, which was also controlled by VPA.II-2 and I-2 suffered from febrile convulsions during childhood, which evolved into afebrile seizures after 10 years old for both of them. Seizure-free was achieved in both of them, by treatment with Phenytoin (PHT) and Carbamazepine (CBZ) respectively. II-4 had focal seizures during the preschool period, and there were no seizures reported since six years old, even without treatment. Another member (II-3) in this family had been suffering from epilepsy since she was less than one-year-old. Unfortunately, seizures were not controlled by CBZ. Early development was normal for her. However, after seizures onset, she had significant retrogression in cognition, and gradually lost the ability of walking and speaking. At 17 years old, she died from sudden unexplained death in epilepsy (SUDEP). Except II-3, other five affected members in the family had normal cognition and motor function, and neurological examinations were unremarkable. Detailed clinical information of the affected individuals in this family was summarized in Table 1 and Additional file 1.

**Table 1:** Clinical features of affected member of *SCN8A* mutation in the pedigree

Patient	Gender	Age (death)	Age of onset	Seizure type	Motor development	Cognitive outcome	EEG (age)	Respond to AEDs
I-2	F	61y	<5 y	Febrile convulsion evolved into afebrile seizure during school-age	Normal	Normal	–	Seizures free from 11 y with PHT
II-2	F	35y	3 y	Febrile convulsion evolved into afebrile seizure after 10 y	Normal	Normal	–	Controlled by CBZ, no seizures for more than 5 y to date
II-3	F	(17y)	<1 y	Focal seizure	regressed	regressed	–	Not controlled by CBZ
II-4	F	27 y	Preschool period	Focal seizure	Normal	Normal	–	Untreated, seizures resolved
III-4	F	6y, 9 mo	6 m	Focal seizure	Normal	Normal	Normal (6 m)	Seizures free from 1-year-old with VPA
III-5	F	2 y, 9 mo	6 m	Focal seizure	Normal	Normal	**–**	No seizures after 1 years old with VPA

*m* months, *y* years, *F* female, *PHT* phenytoin, *VPA* valproic acid, *CBZ* carbamazepine

In addition, a total of 178 sporadic patients having unknown etiological epilepsy were recruited. All of the patients had epilepsy, and met one or more of the following criteria: 1) seizures onset within 6 months, especially within 3 months after birth; 2) intractable epilepsy with frequent seizures; 3) severe electroencephalography (EEG) abnormalities, including hypsarrhythmia, burst-supperession, etc.; 4) severe psychomotor retardation, with or without regression. We excluded the patients with neonatal or infant epileptic seizures caused by metabolic diseases, brain injury during perinatal period, central nervous system infection or other known etiologies.

The detailed clinical information was collected by periodic follow-up, including medical history, family history, curative effects by antiepileptic drugs (AEDs), MRI, EEG, etc.,. Genomic DNA was extracted from peripheral leukocytes. Written informed consent was obtained from the subjects. This study was approved by the Peking University First Hospital Medical Ethics Committee.

### Whole-exome sequencing

Whole-exome sequencing was used to detect the mutated gene in the family. Probes were designed to cover the whole-exome. Sequencing was performed on Ion torrent Proton high-throughput platform (Themofisher) using a paired-end sequencing of 200 bp to screen the mutations. Fast QC v0.10.1 was used to filter out reads of low base quality score. BWA software was used to align the reads to the hg19 genome. Unified Genotyper and HapolotypeCaller in Genome Analysis Tool KIT v3.2 (GATKv3.2) were used to call variants.

### Targeted NGS

Targeted NGS was used to screen gene mutations in 178 sporadic patients with epilepsy of unknown cause. A custom-designed panel capturing the coding exons of 485 genes (Additional file 2) related to epilepsy and intellectual disability, including *SCN8A*, was synthesized using NimbleGenSeqCap Target Enrichment technique. NGS was subsequently performed on Ion torrent Proton high-throughput platform (Themofisher).

### Mutations annotation

All the mutations were confirmed by Sanger sequencing. Rare mutations, whose population frequency was less than 1%, were filtered according to the 1000 Genomes data, ESP6500 population data and ExAC data. Reported pathogenic mutations in HGMD Professional database and Pubmed were marked. The pathogenicity of other rare mutations was annotated by Mutation Taster and PolyPhen-2.

Sanger sequencing was performed to analyze the parental origin of those variants. Then the causative mutations were confirmed according to parental origin of the variants and the clinical features of the patients.

## Results

### Molecular analysis

**Fig. 2:**
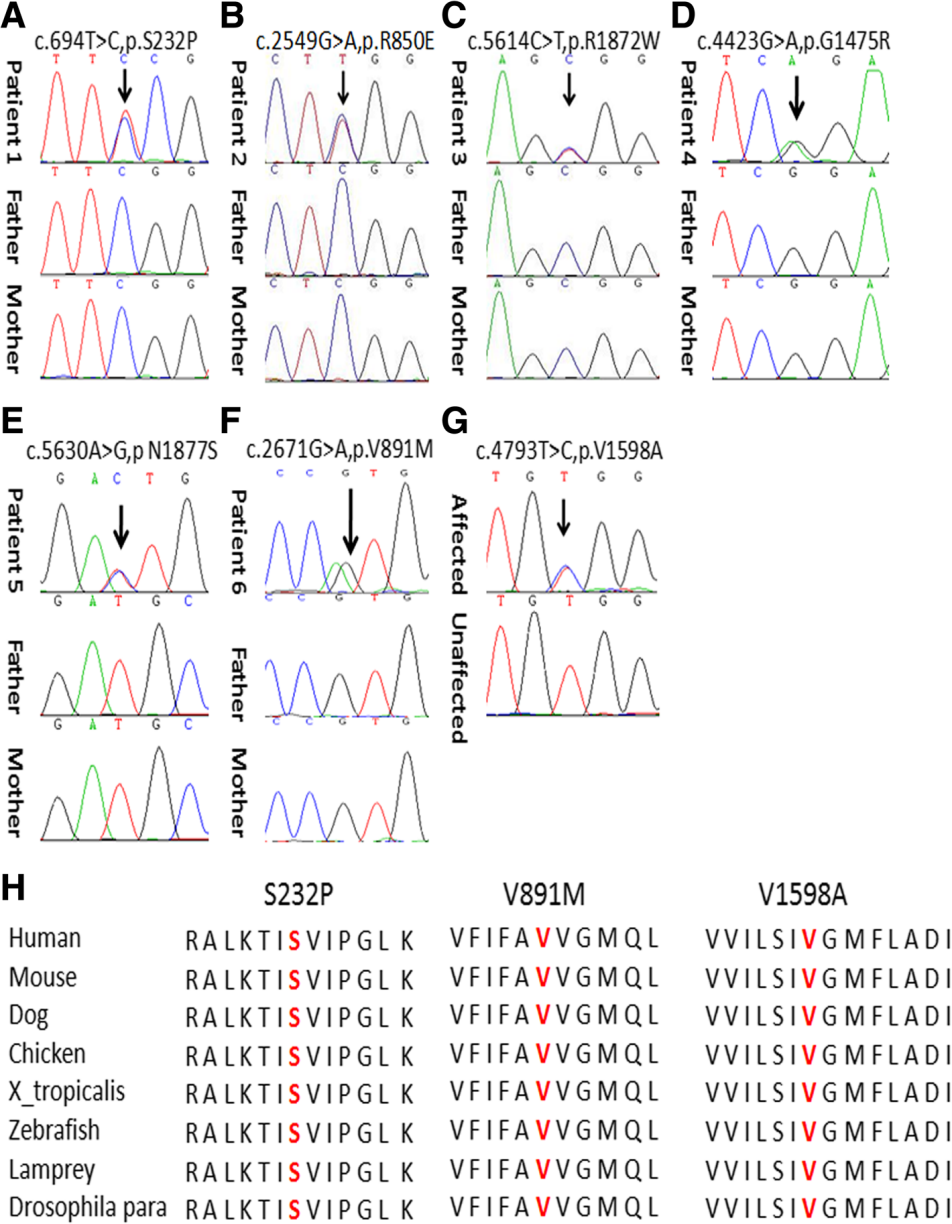
Seven *SCN8A* mutations identified in this study **a**-**g** and conservation of the altered amino acid of three unreported mutations shown in the ClustalW alignments **h**

Five members, including four patients (I-2, II-2, II-4, III-4) and one phenotypic normal member (III-2) in the Chinese family were analyzed by whole-exome sequencing (Fig. 1). Meanwhile, patient III-5 was not born yet. A total of 23 candidate gene mutations were identified (Additional file 3). After III-5 was born and presented with seizures at age of 6 months, Sanger sequencing of these 23 candidate genes was performed on the newborn. Then it was narrowed to 10 candidate genes including *SCN8A* and 9 other genes (Table 2)*.* Among these candidate genes, 7 genes were neither expressed in nervous system, nor reported to be associated with epilepsy or other neurologic disorders. *ANGPT4* has been related to Alzheimer’s disease and vascular dementia. *IMPA1* has been associated with bipolar disorder previously. But the variants of them detected in this family were also frequent in ExAC and 1000 Genomes database. *SCN8A* has been related with EIEE 13, benign infantile epilepsy, and cerebellar ataxia. It is widely expressed in central nervous system and had been reported in patients with epilepsy. The heterozygous missense mutation (c.4793 T > C, p.Val1598Ala) of *SCN8A* identified in this family was not mentioned in any known database (dbSNP138, 1000G, EVS, ExAC). The mutation site was highly conserved from insects to mammals (Fig. 2h), and was predicted as damaging by Mutation Taster and PolyPhen-2 (Table 3). So *SCN8A* was considered to be the causative gene of the family.

**Table 2:** 10 candidate genes identified in the family case and their related disease

Gene	Related disease	Inheritance	Transcript	Mutation	Allele carriers in ExAC	Allele carriers in 1000 G
*SCN8A*	Cognitive impairment with or without cerebellar ataxia; EIEE13; Seizures, benign familial infantile, type 5	AD	NM_014191.3	c.4793 T > C, p.Val1598Ala	0	0
*ANGPT4*	Alzheimer’s disease, vascular dementia	Unknown	NM_015985	c.68A > T, p.Gln23Leu	3	83
*IMPA1*	Mental retardation, type 59	AR	NM_001144878	c.856A > G, p.Ile286Val	2	–
*POP1*	Anauxetic dysplasia, type 2	AR	NM_001145860	c.2861G > A, p.Arg954His	1	–
*RYR3*	Atherosclerosis; HIV Infections	Unknown	NM_001243996	c.12448G > A, p.Asp4150Asn	23	2
*SLC16A3*	Atherosclerosis; Cardiovascular Diseases; Diabetes Mellitus, type 2	Unknown	NM_004207	c.44C > T, p.Ala15Val	600	57
*TDRD7*	Cataract, autosomal recessive, type 36	AR	NM_014290	c.474G > A, p.Met158Ile	14	1
*PKP2*	Arrhythmogenic right ventricular cardiomyopathy, type 9	AD	NM_001005242	c.2200A > G, p.Ile734Val	6	1
*LAMA3*	Epidermolysis bullosa, generalized atrophic benign; Epidermolysis bullosa, junction, Herlitz type; Laryngoonychocutaneous syndrome	AR	NM_198129	c.7462G > A, p.Asp2488Asn	–	–
*C6ORF165*	No related diseases reported	_	NM_001031743	c.274A > G, p.Trp92Ala	96	7

*AD* autosomal dominant, *AR* autosomal recessive, *EIEE 13* Epileptic encephalopathy, early infantile, type 13

**Table 3:** Seven *SCN8A* (NM_014191.3) mutations identified in our study

Patient	Mutation	Chromosome 12: position	Domain	Mutation taster	PolyPhen-2	Reported/Novel
Patient 1	c. 694 T > C, p.Ser232Pro	52,082,621	DIS4	Disease causing	Probably damaging	*Novel*
Patient 2	c.2549G > A, p.Arg850Glu	52,159,459	DIIS4	Disease causing	Probably damaging	Reported [14]
Patient 3	c. 5614C > T, p.Arg1872Trp	52,200,884	C-terminal	Disease causing	Probably damaging	Reported [9, 14]
Pateint 4	c. 423G > A, p.Gly1475Arg	52,184,185	DIIIS6-DIVS1 loop	Disease causing	Probably damaging	Reported [15]
Patient 5	c.5630A > G, p.Asn1877Ser	52,200,900	C-terminal	Disease causing	Possibly damaging	Reported [15, 16]
Patient 6	p.2671G > A, p.Val891Met	52,159,581	DIIS5	Disease causing	Probably damaging	*Novel*
Pedigree	c.4793 T > C, p.Val1598Ala	52,188,423	DIVS3	Disease causing	Probably damaging	*Novel*

The positions of the mutations on chromosome 12 refer to the reference sequence that was retrieved from the NCBI database (build 37)

**Fig. 3:**
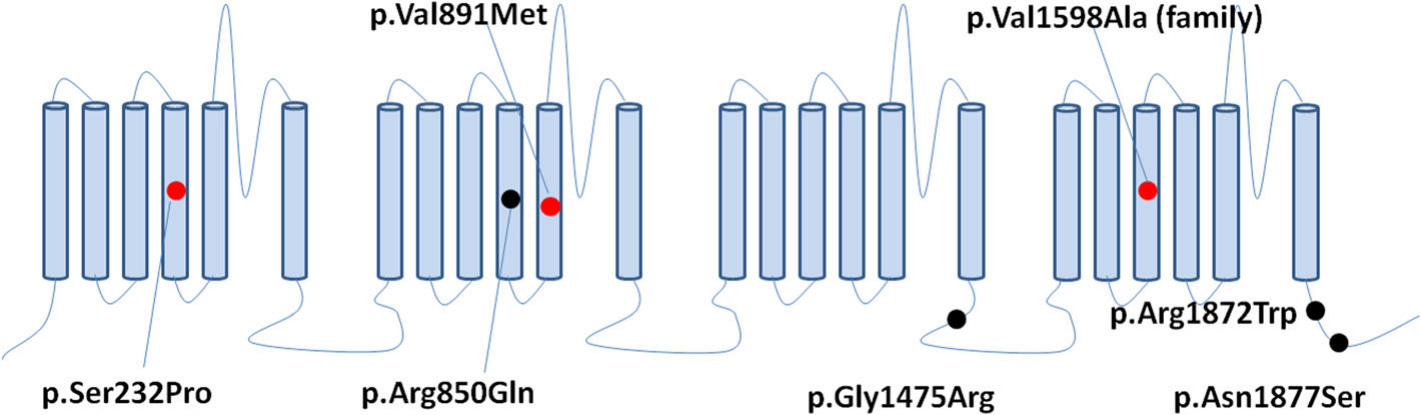
The structure of the human Nav1.6 channel and the location of *SCN8A* mutations identified in this study. Red Dots: Novel; black dots: reported

By targeted NGS, heterozygous mutations in seven genes (*CDKL5, KCNQ2, KCNT1, STXBP1, SCN2A, SCN8A, SLC2A1*) were detected in 59 patients. The gene mutation rate was 33% (59/178). *SCN8A* gene mutations were identified in six patients (Fig. 2a-f). All six mutations were de novo, which were predicted highly damaging the function of Na_v_1.6 channel by Mutation Taster and PolyPhen-2 (Table 2). Four of them have been confirmed pathogenic previously (Table 3). The other two *novel SCN8A* mutations (c.694 T > C, p.Ser232Pro and c.2671G > A, p.Val891Met) are located at the extremely conserved positions (Fig. 2h). A schematic representation of the Na_v_1.6 channel, including the locations of mutations identified in this study, is shown in Fig. 3.

### Clinical and neurophysiological findings

#### Family case

The clinical information of six affected patients was summarized in Table 1.

#### Sporadic cases

Six patients were identified having *SCN8A* mutations, including four males and two females. The clinical features of them were diverse, of whom four presented severe epileptic encephalopathy, whereas the other two had much milder clinical course. The age of epilepsy onset ranged from 2 month to 5 month, averaging at 3.9 months. Focal seizure was the most common semiology, observed in all six of them. Seizure-free was achieved in four of them eventually.

Patient 1 had focal seizures beginning at two months after birth. Seizures attacked 2–3 times every day. He was treated with Topiramate (TPM) at 3 months of age, and achieved a seizure-free period as long as 45 days. At 5 months old, seizures recurred, and the frequency was 10 times per day. Levetiracetam (LEV) was used but without remission. Then VPA was added, and seizure frequency was reduced to 5–6 times per day. TPM was stopped at 8 months old, and Clonazepam (CZP), Oxcarbazepine (OXC) was added at 11 months of age. By treatment with multi-drugs (VPA, OXC, CZP), no seizures were observed since from one year of age. He presented with severe developmental delay and is currently (32 months of age) unable to sit independently.

Patient 2 was discovered to have epilepsy at 3 months of age. The initial seizure type was focal seizure. Seizures attacked 7–8 times per day. He was treated with LEV at 5 months, but no good outcome was reported. At 9 months of age, myoclonic seizures were observed. EEG at that time showed generalized high amplitude multi-spike and slow waves. At age of 1 year and 7 months, LEV was substituted by VPA, and seizure frequency was reduced to 3 times per day. By 1 year and 11 months, Lamotrigine (LTG) was added for treatment, and no seizures were reported after 2 years old. The developmental milestones were far behind his peers, that he could not control his head until 28 months old.

Patient 3 began to have seizures at age of 3.5 months, including focal seizures, secondary generalized seizures, and status epilepticus. It was as frequent as 10–15 times per day. EEG at 1-year-old showed slow background and spikes at right central area during sleep. He remained have seizures at age of one year, even with multi-therapy (LEV, TPM, VPA and OXC). Developmental delays caused concern since from 3 months of age, when poor head control was observed, and there was no apparent improvement throughout the follow-up period (one-year-old). As patient 3 was loss to follow-up, his current status was not clear.

Patient 4 developed epilepsy at 4 months old. The initial seizure type was focal seizures. Generalized seizures were also observed. Seizures attacked 1–2 times per week. She was treated with VPA at 5 months of age, but showed no signs of remission. Then TPM was added at 6 months old. By treatment with VPA and TPM, she had no seizures for 6 months (from 6-month-old to 1-year-old). However, seizures attacked again at 1 year old and the frequency was as high as 1–2 times per day. By that time, CZP was added but with no effects. CBZ was added at 2 years and 2 months old, by which seizure frequency was markedly reduced, and the intermittent phase could be as long as 50 days. EEG was at boundary condition at 2 years of age. She presented with severe hypotonia and couldn’t control head at age of 26 months. After the seizure frequency was reduced, she showed significant developmental progress, that she could control head at 2 years and 5 months.

Patient 5 demonstrated milder phenotype. Epilepsy onset was at 5 months old, with focal seizures followed by secondary generalization. EEG at age of 8 months showed sharp and spike waves in the right frontal during sleeping period, and 3–4 Hz slow-wave complexes in the occipital region during awake period. Seizures attacked 1–2 times per day. VPA and LEV were not effective at age of 8 months. Then OXC was added at that time, no seizure was reported from then on. Developmental milestones were normal before the onset of seizures, with head control at 2 months of age. After seizures onset, her development was slightly delayed that she could not sit independently until 9 months. Patient 5 didn’t show obvious cognition impairment.

Patient 6 had focal seizures starting at 6 months old. Seizures attacked once every month. At 11 months old, he was treated with LEV, but seizures still occurred occasionally. OXC was used at 18 months of age, and since then no seizures were observed. Then two months later LEV was stopped. He presented with normal development and cognition.

The detailed clinical information of the six sporadic cases was summarized in Table 4 and Additional file 1.

**Table 4:** Clinical features of six sporadic cases with *SCN8A* gene mutation

Patient	Sex	Age	Age of onset	Seizure type	Motor development	Cognitive outcome	EEG (age)	MRI	Respond to AED	Hypotonia
Patient 1	M	1 y, 15 m	2 m	Focal seizure	Head control (15 m), unable to sit alone (1 y, 15 m)	Severe ID	Asynchronous bilateral spike, spike and wave discharges, with central, parietal and frontal predominance (3 m)	None	OXC 30 mg/kg/d, VPA 32 mg/kg/d, CZP 0.6 mg/d; no seizure from 1 y	**+**
Patient 2	M	2 y, 8 m	3 m	Focal seizure (3 m), myoclonic seizure (9 m)	Head control (2 y, 4 m)	Severe ID	Generalized high amplitude multi-spike and slow waves (9 m)	Normal (3 m); slight widened cerebellar sulci (8 m)	VPA 42 mg/kg/d, LTG 4.5 mg/kg/d, seizure free from 2 y	**+**
Patient 3	M	3 y, 6 m	3.5 m	Focal seizure, secondary generalized seizure, status epilepticus	Can’t control head at 1-year-old	Severe ID	Diffuse slow waves in the background, spike at right central area during sleep period (1 y)	Normal (3 m)	LEV 29.4 mg/kg, OXC 49.4 mg/kg, TPM 5.9 mg/kg, VPA 23.5 mg/kg; Seizures were not controlled –during follow-up visit (before 1 y)	**+**
Patient 4	F	2 y, 6 m	4 m	Focal seizure (4 m), generalized seizure (2 y, 2 m)	Head control at age of 2 y, 5 m	Severe ID	Boundary condition (4 m)	Normal (2 y)	Seizures frequency reduced by VPA 36 mg/kg/d, TPM 6.8 mg/kg/d, CZP 0.5 mg/d, and CBZ 18 mg/kg/d	**+**
Patient 5	F	1 y	5 m	Focal seizure with generalization	Head control (2 m), sit independently (9 m), stand with assistance (1 y)	Normal	sharp and spike waves in the right frontal during sleep period, and 3–4 Hz slow-wave complexes in the occipital during awake period (8 m)	Normal (7 m)	Seizures were controlled by LEV (48.9 mg/kg/d), VPA (26.7 mg/kg/d), OXC 20 mg/kg/d; no seizures were reported since 8-month-old	**–**
Patient 6	M	1 y, 8 m	6 m	Focal seizure	Head control (3 m), sit alone (6 m), walk (1 y, 4 m)	Normal	Normal EEG (6 m); Boundary condition (1 y, 6 m)	Normal (1 y, 6 m)	Controlled by OXC (30 mg/kg/d), no seizures reported since 18 m	**–**

*m* months, *y* years, *F* female, *M* male, *ID* intellectual disability

## Discussion

A total of seven *SCN8A* mutations were identified, of which three were *novel*. These new patients provide support for delineating the clinical features of patients with *SCN8A* mutations.

*SCN8A* mutations have already been proved responsible for EIEE 13. Generally, patients had seizure onset within 6 months after birth [9]. The seizure semiology was variable, including focal seizures, clonic seizures, tonic-clonic seizures, epileptic spasms, myoclonic and absence seizures, etc. [9, 12]. The majority of affected patients had mild to severe psychomotor retardation. Hypotonia, hypertonia and dystonia were common as well [9]. Patients with *SCN8A* mutations also have a high incidence of SUDEP [13, 14]. In our study, the clinical profiles of four patients (Patient 1–4) were in accordance with the characteristic of EIEE13. Seizures began at a mean age of 3.5 months and were intractable. Focal seizure was the primary seizure type. All of them suffered from severe developmental delay and intellectual disability (Table 4). Hypotonia was also obvious. The other two patients (patient 5, and 6) presented with milder phenotype, which confirmed that diseases caused by *SCN8A* mutations had a broad phenotypic-spectrum.

Four mutations (those of patient 2, 3, 4, and 5) had been described previously. The clinical features of patient 2, 3, and 4 were quite similar as the characterization of patients with each same mutation previously described [12, 14, 15]. The phenotype of patient 5 was much milder. With multi-drugs (LEV, VPA, OXC), no seizures were observed since 8 months old. In addition, she didn’t present obvious cognition impairment, whereas her phenotype was slightly more serious than the patients with the same mutation (c.5630A > G, p. Asn1877Ser) in Anand’s report [16]. Anand et al. reported a family with *SCN8A* mutation, in which the proband and his father had early onset focal epileptic seizures without cognitive or neurological impairment. The seizures were controlled well by mono-therapy, with CZB and PHT, respectively. Additionally, in the report of Parrini et al., patients with the same mutation (c.5630A > G, p. Asn1877Ser) presented with drug resistant focal epilepsy and mild intellectual disability [15]. Nevertheless, this mutation has also been reported to cause more severe phenotype including intractable epilepsy, developmental delay and intellectual disability [16]. This study and previous reports suggest that same mutation in *SCN8A* can lead to a different phenotype.

Patient 6, with mutation (c.2671G > A, p.Val891Met) in *SCN8A*, had a benign clinical course. He presented no psychomotor retardation, and epilepsy was well controlled by mono-therapy. It was the first reported de novo *SCN8A* mutation relating with benign phenotype. Combined with previous reports, our study provided further evidence that *SCN8A* mutations could lead to a wild range of phenotypes.

To date, more than 100 *SCN8A* mutations have been reported, an overwhelming majority of which were de novo [12, 14, 17–29]. Only few inherited *SCN8A* mutations were reported. Trudeau et al. first reported a frame-shift mutation (c.5156_5157delCT, p.Pro1719fs) of *SCN8A* in a family with mental retardation and ataxia, but without epilepsy [11]. Gardella et al. discovered a *SCN8A* mutation (c.4447G > A, p.Glu1483Lys) in 16 affected members of three families with BFIS/ICCA [10]. Anand et al. identified a heterozygous mutation (c.5630A > G, p.Asn1877Ser) of *SCN8A* in a family with early onset epilepsy with no cognitive impairment [16]. The heterozygous mutation (c.4793 T > C, p.Val1598Ala) identified in our Chinese family was the forth reported inherited *SCN8A* mutation. In addition, all patients in this family were female, and *PCDH19* gene mutation was ruled out. The onset age ranged from 6 months to preschool period. Two (I-2 and II-2) of them initialized as febrile convulsion, which evolved into afebrile seizures. The others initialized as focal seizures. Good seizure control was achieved in four of them with mono-therapy, and II-4 was seizure free even without treatment. While seizures were refractory inII-3. At the age of 17 years, she died from SUDEP. Except II-3, the other five affected members had normal cognition and developmental profile. To the best of our knowledge, this is the only family among which different phenotypes presented in the members with the same *SCN8A* mutation. It is proposed that other genetic or non-genetic factors account for the different phenotypes. Unfortunately, as II-3 had passed away, the difference of genetic background between the affected members, which might explain their significant variation on phenotypes, was not available.

The reported mutations are scattered throughout the whole *SCN8A* channel [9]. Among these mutations, Arg^1872^ and Arg^1617^ might be two mutational hotspots, which had been identified in more than ten independent patients [14, 17, 23, 26]. In vitro functional analysis proved that both of the mutations Arg^1872^ and Arg^1617^ impaired the Na_v_1.6 channel transition from open state to inactivated state, resulting in channel hyperactivity [30]. Like Arg^1617^, the mutations c.2549G > A, p.Arg850Glu and c.694 T > C, p.Ser232Pro identified in this study were located in S4 transmembrane segments (Figure 3), which served as the voltage sensors for Na_v_1.6 activation. So, these two mutations might have the similar pathogenic mechanism as mutations at Arg^1617^. They might lead to elevating Na_v_1.6 channel activity by impairing channel inactivation.

Sodium channel blockers may be the rational candidate drugs for the treatment of epilepsy caused by *SCN8A* mutations, including OXC,CBZ, LTG, Phenobarbital (PB), TPM and PHT, etc. [31, 32]. For benign familial infantile epilepsy caused by *SCN8A* mutation, seizures were easily controlled by mono-therapy. While for the epileptic encephalopathy, seizures were not easy to control. Approximately half of patients showed good responses to sodium channel blockers, either as a reduction in seizures frequency or even seizure-free [31, 32]. Up to 1/3 of patients were seizure-free after treatment with antiepileptic drugs [9]. In our study, four sporadic patients were seizure-free by treatment with mono- or multi- antiepileptic drugs. All of them were treated with sodium channel blockers.

## Conclusions

*SCN8A* mutation is not only associated with epileptic encephalopathy, but also can be the pathogenic cause of some benign phenotypes, such as BFIS/ICCA, especially the inherited mutations. Three *novel SCN8A* mutations were identified in this study. The huge difference among the family affected members, and the varied phenotype between patient 5 and patients previously reported with same mutation (c.5630A > G, p.Asn1877Ser), suggested that the same mutation in *SCN8A* can lead to quite different phenotypes. It is necessary to analyze *SCN8A* mutations in patients with early onset epilepsy with or without developmental delay or intellectual disability. The limited clinical evidence suggests that sodium channel blockers may be the good choice for patients with *SCN8A* mutations.

## Additional files

Additional file 1: The record of seizures attack of patients with *SCN8A* mutations. This file included the seizure frequency of patients with *SCN8A* mutations, at different periods. (DOCX 16 kb)

Additional file 2: 485 genes in the targeted-next generation sequencing panel. (PDF 45 kb)

Additional file 3: 23 candidate genes identified in the Chinese family with epilepsy, by whole-exome sequencing. (DOCX 16 kb)

## Abbreviations

AEDs: Antiepileptic drugs
AIS: Axonal initial segment
BFIS: Benign infantile seizures
CBZ: Carbamazepine
CZP: Clonazepam
EEG: Electroencephalography
EIEE: Early infantile epileptic encephalopathy
ICCA: Infantile convulsion and paroxysmal choreoathetosis
LEV: Levetiracetam
LTG: Lamotrigine
OXC: Oxcarbazepine
PB: Phenobarbital
PHT: Phenytoin
SUDEP: Sudden unexpected death in epilepsy
TPM: Topiramate
VGSCs: Voltage-gated sodium channels
VPA: Valproic acid
