# Supplementary material for: SCN8A mutations in Chinese patients with early onset epileptic encephalopathy and benign infantile seizures
Source: BMC Med Genet. 2017 Sep 18;18:104. doi: 10.1186/s12881-017-0460-1 (PMC5604297; doi:10.1186/s12881-017-0460-1)
Supplement: Supplementary file 1 — The record of seizures attack of patients with SCN8A mutations. This file included the seizure frequency of patients with SCN8A mutations, at different periods. (DOCX 16 kb) [file 12881_2017_460_MOESM1_ESM.docx]

**Additional file 1.** The record of seizures attack of patients with *SCN8A* mutations

| **Patient** | **SZ Frequency Now** | **SZ Frequency At Worst** | **SZ Free (Y/N)** | **Date of Last SZ** |
| --- | --- | --- | --- | --- |
| 1 | 0 | 10 times/d | Y | 1 y |
| 2 | 0 | 7-8 times/d | Y | 2 y |
| 3 | Not clear | 10-15 times/d | — | — |
| 4 | 1-2 times/m | 1-2 times/d | N | — |
| 5 | 0 | 1-2 times/d | Y | 8 m |
| 6 | 0 | once/m | Y | 1 y, 6 m |
| I-2 | 0 | Not clear | Y | 11 y |
| II-2 | 0 | Not clear | Y | 30 y |
| II-3 | Died | Not clear | — | — |
| II-4 | 0 | 1-2 times/y | Y | 6 |
| III-4 (P) | 0 | 6 times/d | Y | 1 y |
| III-5 | 0 | 4-5 time/week | Y | 1 y |

Motor skills of six sporadic patients with *SCN8A* mutations

| **Patients** | **Eye Contact** | | **Grasping Objects** | | **Head Control** | | **Sitting** | | **Walking** | | **Running** | | **Speaking Ability** |
| --- | --- | --- | --- | --- | --- | --- | --- | --- | --- | --- | --- | --- | --- |
|  | Y/N | Age acquired | Y/N | Age acquired | Y/N | Age acquired | Y/N | Age acquired | Y/N | Age acquired | Y/N | Age acquired | NonVerbal/Limited Verbalizations/No Problems |
| 1 | Y | 9 m | N | — | Y | 1 y, 3 m | N | — | N | — | N | — | Limited verbalizations |
| 2 | Y | 2 y | N | — | Y | 2 y, 4 m | N | — | N | — | N | — | NonVerbal |
| 3 | NA | — | NA | — | NA | — | NA | — | NA | — | NA | — | NA |
| 4 | Y | 2 y | N | — | Y | 2 y, 5 m | N | — | N | — | N | — | NonVerbal |
| 5 | Y | 2 m | Y | 6 -7m | Y | 2 m | Y | 9 m | N | — | N | — | Limited verbalizations |
| 6 | Y | 3 m | Y | 6-7 m | Y | 3 m | Y | 6 m | Y | 1 y, 4 m | N | — | Limited verbalizations |

Y, yes; N, no; NA, not available; y, year; m, month
