# Supplementary material for: SCN8A mutations in Chinese patients with early onset epileptic encephalopathy and benign infantile seizures
Source: BMC Med Genet. 2017 Sep 18;18:104. doi: 10.1186/s12881-017-0460-1 (PMC5604297; doi:10.1186/s12881-017-0460-1)
Supplement: Supplementary file 2 — 485 genes in the targeted-next generation sequencing panel. (PDF 45 kb) [file 12881_2017_460_MOESM2_ESM.pdf]

Additional file 2. 485 genes in the targeted-next generation sequencing panel

ABAT	*BDNF*	*CPA6*	*GABRA4*	*HSD17B10*	*MIB1*	*PEX5*	*SCN1B*	*ST3GAL3*
ABCB1	*BLVRB*	*CPS1*	*GABRA5*	*HTR1A*	*MMADHC*	*PEX6*	*SCN2A*	*ST3GAL5*
ABCC2	*BRAT1*	*CPT2*	*GABRA6*	*IDH2*	*MOCS1*	*PFKL*	*SCN2B*	*STK11*
ABCC8	*BRD2*	*CRH*	*GABRB2*	*IDS*	*MOCS2*	*PFKM*	*SCN3A*	*STRADA*
ABCG2	*BTD*	*CS*	*GABRB3*	*IER3IP1*	*MOG*	*PHF6*	*SCN3B*	*STXBP1*
ACADM	*C10ORF2*	*CSTB*	*GABRD*	*IL6ST*	*MR1*	*PHGDH*	*SCN4A*	*SUOX*
ACMSD	*C3*	*CTDP1*	*GABRG2*	*IMPA2*	*MSN*	*PHOX2A*	*SCN5A*	*SV2A*
ACO2	*CA1*	*CTSD*	*GABRR2*	*INA*	*MTHFR*	*PIGA*	*SCN7A*	*SYN1*
ACOX1	*CACNA1A*	*CUL4B*	*GAD1*	*IQSEC2*	*MTMR9*	*PIGL*	*SCN8A*	*SYNGAP1*
ACP1	*CACNA1E*	*CXCR1*	*GAMT*	*JRK*	*MTOR*	*PIGN*	*SCN9A*	*SYP*
ACSF3	*CACNA1G*	*CYP11A1*	*GATM*	*JUN*	*MTR*	*PIGO*	*SDHA*	*SYT11*
ACTA2	*CACNA1H*	*CYP3A4*	*GBA*	*KARS*	*MYO5C*	*PIGV*	*SEMA3A*	*SZT2*
ACTB	*CACNA2D2*	*CYP4F11*	*GC*	*KCNA1*	*NAGA*	*PIR*	*SEPSECS*	*TAP1*
ACY1	*CACNB4*	*D2HGDH*	*GCDH*	*KCND2*	*NDE1*	*PKHD1*	*SERPINI1*	*TBC1D24*
ADAR	*CACNG2*	*DBP*	*GCH1*	*KCNE2*	*NDP*	*PLCB1*	*SEZ6*	*TBCE*
ADCK3	*CALHM1*	*DCX*	*GCM2*	*KCNH5*	*NDUFV1*	*PLEC*	*SGCE*	*TBX1*
ADK	*CASK*	*DDC*	*GCSH*	*KCNH6*	*NEDD4L*	*PNKD*	*SHANK3*	*TCF4*
ADORA1	*CASP9*	*DEPDC5*	*GIPC3*	*KCNJ1*	*NGLY1*	*PNKP*	*SHBG*	*THUMPD1*
ADRA1B	*CASR*	*DGCR2*	*GJA1*	*KCNJ10*	*NHLRC1*	*PNPO*	*SLC12A1*	*TICAM1*
ADSL	*CBS*	*DGKD*	*GJD2*	*KCNJ11*	*NIPA2*	*POLG*	*SLC12A6*	*TK2*
AFG3L2	*CCDC88C*	*DHFR*	*GLB1*	*KCNJ6*	*NOS1*	*POLG2*	*SLC16A1*	*TLR3*
AGTR2	*CCL3*	*DIAPH3*	*GLDC*	*KCNK3*	*NPTX2*	*PPP1R3C*	*SLC17A5*	*TMEM67*
ALDH4A1	*CCL4*	*DLX2*	*GLRA1*	*KCNK9*	*NPY*	*PPT1*	*SLC17A7*	*TNF*
ALDH5A1	*CCM2*	*DLX5*	*GLRB*	*KCNMA1*	*NRXN1*	*PRF1*	*SLC19A3*	*TNS1*
ALDH7A1	*CDK5*	*DNM1*	*GLUD1*	*KCNQ2*	*NSDHL*	*PRICKLE1*	*SLC1A1*	*TPP1*
ALG13	*CDKL5*	*DPM1*	*GM2A*	*KCNQ3*	*NSF*	*PRICKLE2*	*SLC1A2*	*TPRXL*
AMACR	*CHD2*	*DPYS*	*GOSR2*	*KCNQ4*	*NTRK1*	*PRNP*	*SLC1A3*	*TREX1*
AMT	*CHGA*	*DYRK1A*	*GPHN*	*KCNT1*	*NTRK2*	*PRODH*	*SLC20A2*	*TRMT44*
ANK3	*CHRFAM7A*	*EEF1A2*	*GPR56*	*KCNV2*	*OPA1*	*PRRT2*	*SLC25A12*	*TRMU*
ANKRD11	*CHRNA2*	*EFHC1*	*GPR98*	*KCTD7*	*OPHN1*	*PSAP*	*SLC25A13*	*TRPM6*
AP4E1	*CHRNA4*	*EFHC2*	*GPX1*	*KDM5C*	*OPRM1*	*PSAT1*	*SLC25A22*	*TSC1*
AQP4	*CHRNA5*	*EGF*	*GRIA3*	*KLK1*	*OTX2*	*PSEN1*	*SLC2A1*	*TSC2*
ARC	*CHRNA7*	*EGR1*	*GRIK1*	*KRIT1*	*PAFAH1B1*	*PSEN2*	*SLC35A3*	*TSEN2*
ARG1	*CHRNB2*	*EGR3*	*GRIK2*	*L1CAM*	*PAH*	*PTEN*	*SLC35C1*	*TSEN34*
ARHGEF15	*CLCN2*	*EHMT1*	*GRIN1*	*LAMA2*	*PALLD*	*PTH*	*SLC46A1*	*TSEN54*
ARHGEF9	*CLCN4*	*EIF2AK3*	*GRIN2A*	*LAMB1*	*PANX1*	*PTS*	*SLC4A3*	*TSPO*
ARSA	*CLCNKB*	*ELOVL4*	*GRIN2B*	*LBR*	*PARK2*	*QDPR*	*SLC6A1*	*TUBA1A*
ARX	*CLEC18A*	*ELP4*	*GRM1*	*LGI1*	*PC*	*RAB39B*	*SLC6A11*	*TUBA8*
ASAH1	*CLIC2*	*EMX2*	*GRN*	*LGI2*	*PCDH19*	*RANBP2*	*SLC6A13*	*TUBB2B*
ASCL1	*CLN3*	*EN2*	*HAX1*	*LGI4*	*PDCD10*	*RBFOX1*	*SLC6A19*	*TUBGCP6*
ASIC1	*CLN5*	*EPM2A*	*HCCS*	*LIAS*	*PDHA1*	*RBFOX3*	*SLC6A8*	*U2AF1*
ASPA	*CLN6*	*EPM2AIP1*	*HCFC1*	*LIFR*	*PDHX*	*RBPJ*	*SLC9A2*	*UBC*
ASPM	*CLN8*	*EXT1*	*HCN1*	*LMBRD1*	*PDXK*	*RCN2*	*SLC9A3*	*UBE2A*
ASS1	*CNP*	*FADD*	*HCN2*	*MANBA*	*PDYN*	*RDX*	*SLC9A6*	*UBE3A*
ATIC	*CNR1*	*FARS2*	*HCN4*	*MAOB*	*PEX1*	*RELN*	*SLC9A9*	*UBR5*
ATN1	*CNTF*	*FKTN*	*HDAC4*	*MAP2*	*PEX10*	*RHAG*	*SMARCA2*	*VAMP2*
ATP1A2	*CNTN2*	*FLNA*	*HEPACAM*	*MAPK3*	*PEX12*	*RHOA*	*SMARCA4*	*VLDLR*
ATP1A3	*CNTNAP2*	*FOLR1*	*HEXA*	*MBD5*	*PEX13*	*RMND1*	*SNIP1*	*VPS13A*
ATP2A2	*COG6*	*FOS*	*HEXB*	*MBTPS2*	*PEX14*	*RNASEH2A*	*SOBP*	*WARS*
ATP6AP2	*COG8*	*FOXG1*	*HLCS*	*ME2*	*PEX16*	*RNF213*	*SPAST*	*WDR45*
ATP6V0A2	*COL4A1*	*FUCA1*	*HMGCL*	*MECP2*	*PEX19*	*ROGDI*	*SPR*	*ZEB2*
ATP7A	*COL6A2*	*GABBR1*	*HNRNPU*	*MED17*	*PEX2*	*RPS6KA3*	*SPTAN1*	*GABRB1*
AUH	*COQ2*	*GABBR2*	*HOXA1*	*MEF2C*	*PEX26*	*SCARB2*	*SRGAP2*	*SLC12A5*

BCKDK	*COQ9*	*GABRA1*	*HPD*	*MFSD8*	*PEX3*	*SCN1A*	*SRPX2*
