# Supplementary material for: SCN8A mutations in Chinese patients with early onset epileptic encephalopathy and benign infantile seizures
Source: BMC Med Genet. 2017 Sep 18;18:104. doi: 10.1186/s12881-017-0460-1 (PMC5604297; doi:10.1186/s12881-017-0460-1)
Supplement: Supplementary file 3 — 23 candidate genes identified in the Chinese family with epilepsy, by whole-exome sequencing. (DOCX 16 kb) [file 12881_2017_460_MOESM3_ESM.docx]

**Additonal file 3.** 23 candidate genes identified in the Chinese family with epilepsy, by whole-exome sequencing**.**

| **Candidate genes** | **Transcript** | **Mutation** |
| --- | --- | --- |
| *INADL* | [NM_176877](http://www.ncbi.nlm.nih.gov/nuccore/NM_176877) | c.188A>C, p.Gln63Pro |
| *RPTN* | [NM_001122965](http://www.ncbi.nlm.nih.gov/nuccore/NM_001122965) | c.460A>G, p.Arg154Gly |
| *WDR35* | [NM_001006657](http://www.ncbi.nlm.nih.gov/nuccore/NM_001006657) | c.725A>G, p.Glu242Gly |
| *RNF212* | [NM_001193318](http://www.ncbi.nlm.nih.gov/nuccore/NM_001193318) | c.721T>G , p.Ser241Ala |
| *C6orf165* | [NM_001031743](http://www.ncbi.nlm.nih.gov/nuccore/NM_001031743) | c.274A>G, p.Trp92Ala |
| *XPO7* | [NM_015024](http://www.ncbi.nlm.nih.gov/nuccore/NM_015024) | c.2419A>G, p.Trp808Ala |
| *IMPA1* | [NM_001144878](http://www.ncbi.nlm.nih.gov/nuccore/NM_001144878) | c.856A>G, p.Ile286Val |
| *POP1* | [NM_001145860](http://www.ncbi.nlm.nih.gov/nuccore/NM_001145860) | c.2861G>A, p.Arg954His |
| *TDRD7* | [NM_014290](http://www.ncbi.nlm.nih.gov/nuccore/NM_014290) | c.474G>A, p.Met158Ile |
| *ARMC3* | [NM_173081](http://www.ncbi.nlm.nih.gov/nuccore/NM_173081) | c.776A>G, p.Asn259Ser |
| *EPC1* | [NM_025209](http://www.ncbi.nlm.nih.gov/nuccore/NM_025209) | c.1298T>C, p.Leu433Ser |
| *PCGF6* | [NM_032154](http://www.ncbi.nlm.nih.gov/nuccore/NM_032154) | c.58G>A, p.Ala20Trp |
| *XPNPEP1* | [NM_020383](http://www.ncbi.nlm.nih.gov/nuccore/NM_020383) | c.747G>A, splicing |
| *GPAM* | [NM_001244949](http://www.ncbi.nlm.nih.gov/nuccore/NM_001244949) | c.515G>T, p.Arg172Met |
| *PKP2* | [NM_001005242](http://www.ncbi.nlm.nih.gov/nuccore/NM_001005242) | c.2200A>G, p.Ile734Val |
| *C12orf40* | [NM_001031748](http://www.ncbi.nlm.nih.gov/nuccore/NM_001031748) | c.985G>C, p.Asp329His |
| *SCN8A* | NM_014191.3 | c.4793T>C, p.Val1598Ala |
| *RYR3* | [NM_001243996](http://www.ncbi.nlm.nih.gov/nuccore/NM_001243996) | c.12448G>A, p.Asp4150Asn |
| *MRC2* | [NM_006039](http://www.ncbi.nlm.nih.gov/nuccore/NM_006039) | c.1220A>G, p.Lys407Arg |
| *SMARCD2* | [NM_001098426](http://www.ncbi.nlm.nih.gov/nuccore/NM_001098426) | c.532A>T, p.Met178Leu |
| *LAMA3* | [NM_198129](http://www.ncbi.nlm.nih.gov/nuccore/NM_198129) | c.7462G>A, p.Asp2488Asn |
| *SLC16A3* | [NM_004207](http://www.ncbi.nlm.nih.gov/nuccore/NM_004207) | c.44C>T, p.Ala15Val |
| *ANGPT4* | [NM_015985](http://www.ncbi.nlm.nih.gov/nuccore/NM_015985) | c.68A>T, p.Gln23Leu |
